# Short and long-term health consequences of the 2013 Sarin attack in Ghouta, Syria: a retrospective descriptive study of civilian survivors

**DOI:** 10.1038/s41598-026-47135-z

**Published:** 2026-04-02

**Authors:** MHD Bahaa Aldin Alhaffar, Luluwa Zarzar, Anneli Eriksson, Salim Namour

**Affiliations:** 1 Department of Global Public Health, Disaster Medicine Research Group, Karolinska Institute, Stockholm, Sweden; 2 European Centre for Diseases Prevention and Control, Diseases Programs, Stockholm, Sweden; 3 Damascus University, Damascus, Syria; 4 Association of Victims of Chemical Weapons in Syria, Damascus, Syria

**Keywords:** Sarin gas, Chemical weapons, Ghouta attack, Syria, Survivors, Conflict, Public health, Health care, Medical humanities, Psychology, Psychology

## Abstract

On August 21, 2013, sarin gas was used as a weapon of war in multiple areas of Ghouta, rural Damascus, Syria, in the largest confirmed chemical weapons attack against civilians in the 21st century. Despite the scale of the attack, little is known about the long-term medical symptoms experienced by survivors. This study documents the lived experiences and the short- and long-term physical and psychological consequences of sarin exposure among civilian survivors more than a decade after the attack. We conducted a retrospective qualitative descriptive study using thematic analysis based on semi-structured narrative interviews with survivors of the 2013 Ghouta chemical attacks. Participants were selected based on confirmed exposure and willingness to provide detailed accounts of their health status, experiences, and healthcare-seeking behavior. Thematic analysis was used to identify recurrent patterns and outcomes across different domains. Participants reported several medical symptoms ranging in severity, including chronic respiratory issues, neurological complaints, fatigue, and sensory disturbances. The psychological impact was universal, with widespread reports of anxiety, nightmares, panic attacks, and social withdrawal. Most participants experienced significant disruption to their quality of life and described barriers to accessing healthcare. The long-term consequences of sarin exposure in Ghouta extend far beyond the initial event, affecting nearly every aspect of survivors’ lives. These findings suggest a substantial long-term burden of self-reported physical and psychological symptoms among survivors. They highlight the need for sustained medical, psychological, and social support, and underscore the importance of integrating survivor perspectives into post-conflict recovery and health system responses.

**Supplementary Information:**

The online version contains supplementary material available at 10.1038/s41598-026-47135-z.

## Background

Since the onset of the Syrian conflict in March 2011, the country has experienced a prolonged humanitarian crisis with substantial impacts on population health and displacement^1^. The civilian demonstrations rapidly escalated into a protracted and complex armed conflict, reshaping the country’s demographic, political, and health landscapes^2^. Over the past fourteen years, the Syrian conflict has been characterized by widespread violence, mass displacement, and severe human rights violations^3,4^. The use of chemical weapons in civilian areas represents a serious violation of international humanitarian norms and has resulted in substantial physical and psychological harm^5^.

Between 2012 and 2019, several attacks using chemical weapons has been documented, even though the exact number varies between different sources^6,7^, the number of attacks exceeded hundreds, with civilians comprising 97.6% of the reported fatalities^5^. Among the most widely documented incidents were the sarin gas attack in Al-Ghouta, rural Damascus in 2013^7^, and the Khan Sheikhoun attack in 2017^7^. Investigations by the Organization for the Prohibition of Chemical Weapons (OPCW) confirmed the use of prohibited agents, including sarin, chlorine, and sulfur mustard. These attacks not only caused immediate mass casualties but also inflicted long-term neurological, respiratory, and psychological harm on survivors^7^.

The Ghouta attack, in particular, marked a critical turning point in the Syrian conflict. On August 21, 2013, sarin-filled rockets struck multiple locations in the Damascus suburbs—including Zamalka, Ein-Tarma (East Ghouta), and Moadamiya (West Ghouta)—killing over 1,300 people (the exact number is different between different reports) and leaving thousands with chronic health complications^7^. This incident represented the largest-scale use of nerve agents since the Iran–Iraq War (1980–1988) and prompted significant international response and investigation^8^. Under diplomatic pressure, Syria acceded to the Chemical Weapons Convention (CWC) and dismantled its declared stockpile^9^. Despite these measures, chemical attacks continued, with chlorine gas gradually replacing sarin as the most frequently used agent. However, geopolitical constraints and limitations in investigative access have impeded accountability efforts and delayed systematic assessment of long-term health consequences^7,9^.

Sarin (GB, O-isopropyl methyl phosphorofluoridate) was the primary nerve agent used in the Ghouta attack^5^. It is a highly toxic organophosphate that works by irreversibly blocking acetylcholinesterase (AChE), the enzyme responsible for breaking down the neurotransmitter acetylcholine (ACh) at nerve ending^10^. The resulting accumulation of ACh leads to overstimulation of the autonomic, neuromuscular, and central nervous systems. Symptoms include excessive secretions, difficulty breathing, slowed heart rate, and pinpointing pupils. Muscle twitching, weakness, and respiratory paralysis may follow, while effects on the brain can trigger seizures, confusion, coma, and, ultimately, death—most often from respiratory failure due to blocked airways and muscle paralysis^7,10^.

While the acute effects of sarin exposure are well documented, the long-term health consequences remain poorly understood—particularly in Syria, where mass displacement and years of repression obstructed systematic research^11^. Frontline medical reports point to persistent neurological damage, chronic respiratory illness, and psychological trauma among survivors, yet little is known about how these conditions shape health-seeking behaviours and access to care^11^. Political restrictions and insecurity further limited independent investigation, leaving critical medical and scientific gaps^12^.

Despite the scale and gravity of these attacks, documentation has been limited. Limited access to affected areas, restrictions on independent investigation, and challenges in evidence preservation hindered accountability efforts and delayed recognition of survivors’ health needs. Since late 2024, changes in the political and security context have created new opportunities for field-based research and documentation of previously under-studied health outcomes^13^. This study aims to advance the understanding of long-term health consequences among survivors of chemical weapon exposure in Ghouta.

## Aim of the research

This study aims to investigate the lived experiences, psychological impact, and the immediate, short, and long-term medical symptoms among the survivors of Al-Ghouta chemical attack.

## Methodology

### Study design

This research is a retrospective qualitative descriptive study using thematic analysis focusing on the survivors of Al-Ghouta attack. Survivors were defined as individuals who directly experienced and survived the 21 August 2013 Al-Ghouta chemical attack, were physically present in the affected area at the time of the event, and experienced immediate sensory exposure and/or acute health effects consistent with chemical exposure. A qualitative approach was selected to capture long-term health experiences and outcomes that are not adequately documented in clinical records. The study followed STROBE guidelines for reporting of observational studies^14^.

### Study settings and location

**Fig. 1:**
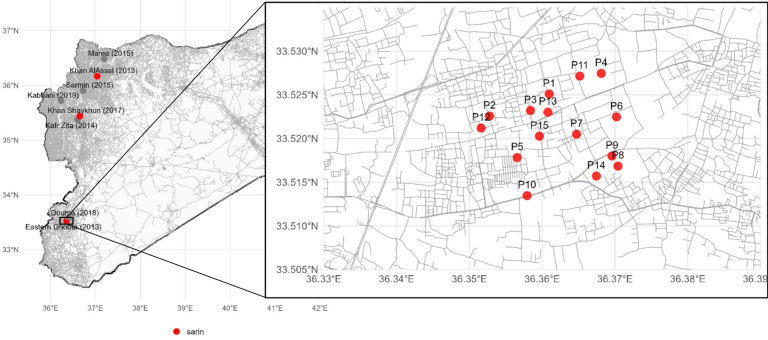
Major chemical weapon attacks in Syria between 2013–2019. Map (left): Syria map generated in R studio using open source data, open street map, Humanitarian data exchange, available from: Syria Roads (OpenStreetMap Export) | Humanitarian Dataset | HDX. Zoomed Map (right): zoomed in map to rural Damascus generated in R studio using open source data, representing Al-Ghouta attacks using sarin on the 21 st of august 2013, visualization of the approximate location based on the details provided from the Association of the victims of chemical weapons Home - Association of Victims of Chemical Weapons. Red dot: chemical weapon attack using Sarin, Gray dot: chemical weapon attacks using other agent (sulfur mustard or chlorine).

Between 2013 and 2019, the number of confirmed chemical weapon attacks in Syria exceeded 300, involving various toxic agents including sarin, chlorine, and sulfur mustard^5^. These attacks caused hundreds of fatalities and thousands of injuries, primarily affecting civilian populations. Among them, the Ghouta sarin attack stands out as the largest in both scale and lethality, and therefore, was the focus on this study. Figure 1 represents the major attack in Syria between 2013 and 2019 with zoomed in map on the attack of 2013 in Ghouta.

This study was conducted in the aftermath of the fall of the Assad regime on December 8, 2024 a contextual change that enabled improved access for documentation and investigation of long-term health effects following chemical weapon exposure, including the long-term effects of chemical weapons. For over a decade, efforts to study these consequences were largely obstructed by systemic denial, targeted repression, and the destruction of evidence. The collapse of the regime has not only allowed survivors to speak more openly but has also facilitated field-based research efforts in areas previously inaccessible to investigators and human rights workers.

Fieldwork was carried out in locations directly affected by the 2013 Ghouta chemical attacks, specifically the neighbourhoods of Zamalka and Ein-Tarma in Eastern Ghouta and Moadamiya in Western Ghouta, Damascus Suburbs Governorate. These areas, situated approximately 16 km apart, were struck in the early hours of August 21, 2013, by rockets filled with sarin gas. According to survivor accounts and OPCW reports, the Zamalka and Ein Tarma strikes occurred between 2:00 and 3:00 AM, while Moadamiya was targeted closer to 5:00 AM^15^.

### Participant inclusion criteria and sampling

Participants were selected using purposive sampling from a pre-existing, independently verified survivor registry maintained by the Association of Victims of Chemical Weapons (AVCW). This registry includes individuals confirmed to have been present in areas affected by the 21 August 2013 Ghouta sarin attack. The research team did not generate the initial survivor list but relied on this database to identify potential participants, after which study-specific inclusion criteria were applied and eligible individuals were contacted. Inclusion criteria required that participants were^1^physically present in the affected areas of Zamalka, Ein Tarma, or Moadamiya at the time of the attack^2^; directly experienced the event firsthand, defined as being present during the attack and experiencing immediate sensory exposure and/or acute health effects; and^3^ were in close proximity to a confirmed impact site. Participant eligibility was confirmed by AVCW, and recruitment was further supported by a trusted field responder and survivor based in Zamalka who facilitated introductions through local networks. Third-person accounts were not included as independent data; references to health outcomes of participants’ children or dependents were included only when reported by the participant as a primary caregiver and treated as contextual information rather than standalone testimonies.

Given the qualitative design and use of narrative interviews, no formal sample size calculation was conducted. The final sample consisted of 14 semi-structured narrative interviews were the participants were asked to tell his/her story without interfering but only directing towards the overall themes of the study. Participants were selected based on verified exposure history to the 21 August 2013 Ghouta sarin attack, as determined by location and timing, as well as availability and willingness to participate. Data collection continued until thematic saturation was reached—that is, when no new information or codes were emerging and the interviews began to yield repetitive themes.

### Interview guide development and data collection

Data collection was guided by a semi-structured interview guide developed specifically for this study. A narrative interview technique was employed, allowing participants to share their experiences freely and with minimal interviewer intervention.

The interview guide was informed by existing literature on chemical weapons exposure, toxicological outcomes, and trauma-informed qualitative research^16^. It was designed to elicit open-ended narratives while maintaining structure around key domains: demographic background, prior medical history, the day of the attack, short- and long-term health symptoms, survival strategies on the day of the attack, and perceived impacts on others (e.g., children, reproductive outcomes). The guide was first drafted in English, then professionally translated into Arabic to ensure cultural and contextual appropriateness^17^. A pilot focus group was conducted with representative of AVCW to test the relevance, clarity, and emotional safety of the questions.

Interviews were conducted using trauma-informed research principles to minimize the risk of re-traumatization and to prioritize participant safety and autonomy^16^. Interviewers received prior training on trauma-sensitive interviewing, including recognizing signs of psychological distress, using non-leading and non-judgmental language, and allowing participants to control the pace and depth of their narratives^18^. Participants were informed that they could pause, skip questions, or terminate the interview at any point without providing a reason. During interviews, distress was actively monitored, and interviewers avoided probing into graphic details unless participants voluntarily introduced them.

Interviews were conducted between January and April 2025 during field visits to previously targeted locations. All interviews were conducted in Arabic, in private and respectful settings chosen by the participants. Depending on logistical constraints and participant preference, all interviews were held in person, in the same locations were the chemical attacks happened, providing direct contextual relevance to the data collected. Each session began with a written informed consent process, ensuring participants understood the study’s purpose, the voluntary nature of participation, and their rights, including the right to withdraw at any time. Interviews lasted between 20 and 50 min.

All transcripts and audio files were stored in encrypted, password-protected drives accessible only to the research team, in accordance with GDPR-compliant data protection standards. A summary of the open-ended prompts used to initiate and guide the narrative interviews is provided in Supplementary Table S1.

### Data analysis

All interviews were audio-recorded, transcribed verbatim in Arabic, and anonymized during transcription to ensure confidentiality. The transcriptions were then reviewed for accuracy and completeness by the research team and translated to English. A thematic analysis approach was applied, following Braun and Clarke’s six-phase framework: familiarization with the data, generation of initial codes, searching for themes, reviewing themes, defining and naming themes, and producing the report^19^.

Initial coding was performed manually in English by two independent researchers trained in qualitative methods. Codes were derived both inductively from the data and deductively from the study objectives. Following the initial coding round, the researchers conducted a cross-checking process to ensure consistency and reliability. Discrepancies in code interpretation were resolved through iterative discussion and consensus, and in cases of ambiguity, a third researcher was consulted.

Following initial coding, codes were compared across interviews and grouped based on conceptual similarity. Related codes were clustered into preliminary themes, which were then reviewed and refined through iterative discussion among the research team to ensure internal coherence and clear distinction between themes. Final themes were defined when they consistently captured recurring patterns across participant narratives and aligned with the study objectives.

A process of recoding was conducted after initial theme development, allowing for the refinement of code categories and the consolidation of overlapping or conceptually similar codes. Themes were then grouped under broader domains reflecting the lived experiences of chemical attack survivors, including recognition of exposure, short and long-term symptoms, health-seeking behaviour, psychosocial impact, and barriers to care.

During analysis of the interviews, medical symptoms were categorized into 1-immediate, 2-short-term, and 3-long-term medical symptoms based on participant narratives. Immediate symptoms occurred directly or within the first hour of exposure; short-term symptoms lasted within hours to two weeks; and long-term effects persisted for months to years. The analysis was conducted manually to retain close engagement with the narrative structure and context. Throughout the process, reflexivity was maintained through analytic memos and debriefing sessions among the research team. Thematic saturation was confirmed when no new codes or themes emerged from additional interviews, supporting the adequacy of the final sample.

Following the completion of the thematic analysis, a focus group discussion was held with members of both the current and former leadership of the Association of Victims of Chemical Weapons (AVCW). The aim was to present and validate the preliminary findings, clarify any inconsistencies, and ensure contextual relevance. Feedback received from AVCW was reviewed systematically, and all relevant insights were incorporated into the final analysis and presentation of results. As data were based on participant narratives, all health outcomes reported in this study represent self-reported symptoms or observed experiences rather than clinically confirmed signs.

### Bias

As with all retrospective studies relying on self-reported data, this study is subject to inherent biases—particularly recall bias and information bias. To mitigate these, a purposive sampling strategy was employed, and only verified survivors—identified and cross-checked through the Association of Victims of Chemical Weapons (AVCW)—were included. Interviews were guided by a semi-structured tool with neutral, open-ended prompts to minimize interviewer influence and enhance the reliability of recollections. Additionally, consistency across participant narratives served as a further internal validity check.

### Ethical considerations

Ethical approval for the study was obtained from the Association of Victims of Chemical Weapons (AVCW), which also served as the data custodian under a formal data-sharing agreement. This agreement outlined strict adherence to confidentiality, data protection, and participant rights.

Each participant provided written informed consent prior to the interview, after receiving detailed information about the study’s aims, methodology, potential risks, and safeguards. Participants were given space to ask questions and were informed of their right to withdraw at any time without consequence. All interviews were conducted in private settings, and data were anonymized during transcription.

Given the trauma-associated nature of the subject matter, interviews were conducted using a trauma-informed approach. Researchers were trained to recognize signs of psychological distress, and interviews were paused or terminated when participants showed discomfort or emotional triggering. This ethical framework aimed to ensure that participants’ dignity, safety, and autonomy remained central throughout the research process.

## Results

### Descriptive analysis of the interviews

This study is based on a total of 14 face-to-face interviews conducted with survivors of the chemical weapons attack that took place in Al-Ghouta, Zamalka. Participants ranged in age from 21 to 60 years, with a mean age of 39. The gender distribution included eight females and six males. In terms of proximity to the chemical attack site, participants reported varying distances, with the closest being approximately 10 m and the furthest up to 100 m away. Most participants were within a 30 to 80-meter radius of the impact zone, likely resulting in a significant exposure to the chemical agents. All participants described that they were healthy and did not any medical symptoms before the attack.

Table 1 presents the main thematic findings from the study. Five overarching themes were identified through the analysis: exposure to the chemical attack and lived experience; immediate medical response and survival strategies; short-term medical symptoms; long-term medical consequences; and psychological impact. Each theme encompassed several subthemes reflecting the complex, layered nature of survivors’ experiences—ranging from sensory and social cues during the attack, to improvised medical responses, to chronic physical and psychological sequelae that persist more than a decade later.

**Table 1:** Summary of study themes, main results and impact of the victims and survivals of the chemical weapons attack.

Themes	Subtheme
Exposure to Chemical Attack and lived experience	- Realizing the chemical attack - Sensory cues - Social cues - Seeking health care - Alerting others
Immediate medical response, treatment, and survival strategies	- Improvised masks - Civilian rescue - Atropine use - Transportation of victims - Seeking shelters - Makeshift tools
Immediate and Short-term medical symptoms	- Ophthalmological problems - Respiratory symptoms - Gastrointestinal (GI) symptoms - Neurological symptoms
Long term medical symptoms	- Cardiologic issues - Chronic respiratory issues - Gastrointestinal (GI) distress - Immune dysfunction - Neurologic problems - Ophthalmological problems - Reproductive Health Disorder - Weakness
Psychological impact	- Self-reported psychological symptoms - Bereavement - Grief - Displacement trauma - Loss of family

### Exposure to chemical attack and lived experience

In the initial phase of the interview, participants were asked to recount their experiences during the attack. Nearly all (13 of 14) described the event as sudden and disorienting, with no official warnings or alerts. Most survivors (9 of 14) identified the threat through sensory cues—unusual smells, lack of typical explosion sounds, and visual anomalies. Others (5 of 14) recognized the attack only after observing the reactions of neighbours or community members. The process of realization was gradual, unfolding as a series of sensory, spatial, and social signals rather than clear communication. In this vacuum of structured alerts, people relied on their instincts and one another to interpret the unfolding event.

*“Its smell was like rotten apples mixed with vinegar… we smelled it and started showing symptoms.” — Participant 2*.

*“We heard a missile without an explosion*,* and black smoke rose.” — Participant 12*.

*“The closest missile to my house was about 100 meters. Its sound was faint and unlike normal shells.” — Participant 4*.

*“There was black smoke*,* and it looked different from a typical bombing.” — Participant 11*.

*“We woke up to the neighbours shouting*,* ‘chemical attack!’ We didn’t understand what was happening. Everyone was shouting and crying.” — Participant 12*.

*“The neighbours knocked on our door yelling ‘chemical attack!’ and we could feel something was wrong with the air.” — Participant 13*.

*“At 1:45 AM*,* we were using our radios and performing rescue work. Suddenly*,* around 15 missiles fell. The first three [missiles] didn’t explode in a normal way. When we went to the site*,* there was a strange smell*,* and one of the people there started convulsing and foaming at the mouth.” — Participant 1*.

*“We asked our team on the radio what kind of missiles these were—this wasn’t normal.” — Participant 1*.

### Immediate medical response, treatment, and survival strategies

In the absence of organized emergency infrastructure, survivors of the Ghouta chemical attack relied on improvised and community-driven responses. All participants reported using household items—such as vinegar, water, cloth, and onion peels—as makeshift gas masks. Eight out of 14 described the use or administration of atropine, though access was uneven and often improvised. Transport to medical facilities was primarily civilian led, with survivors either carried by neighbours or taken by volunteer drivers to overwhelmed field hospitals. These spontaneous efforts reflect the urgent and chaotic environment in which survival depended on shared knowledge, peer support, and the creative use of limited resources.

*“We went to the rooftop and tore a piece from my son’s shirt. We dipped it in vinegar and water and put it on our faces as masks.” — P13*.

*“We used a mask with a piece of cotton soaked in vinegar and water. We put onion peels inside it*,* just like we heard from others—it might help.” — P11*.

*“I opened oxygen tanks and administered atropine. I was injecting people*,* trying to do anything I could.” — P10*.

*“They took us to Kafr Batna. My husband kept helping others the whole time*,* even though he was exposed himself.” — P12*.

*“Medical volunteers were injecting people with atropine… local health workers were giving treatment*,* even in houses.” — P4*.

*“Ambulances came from Arbin*,* from Douma*,* from across Ghouta. But it was too much… there were too many cases at once.” — P3*.

### Immediate and short-term medical symptoms

All participants described a sudden onset of acute medical symptoms during the chemical attack, with excessive salivation and foaming reported by 12 out of 14, muscle convulsions by 11, respiratory distress by 11, ophthalmologic symptoms such as burning or blurred vision by 12, and loss of consciousness by 9. These symptoms began within seconds to minutes after exposure and were multisystemic in nature, affecting respiratory, neurological, and visual systems simultaneously. The rapid progression of effects contributed to a sense of helplessness and panic, especially in the absence of medical support (Table 2).

*“One person convulsed and started foaming at the mouth—we knew then it was chemical.” — P1*.

*“My whole body started shaking uncontrollably. My eyes hurt. I felt like my body wasn’t mine anymore.” — P 13*.

*“The light looked like a thousand suns. Everything was spinning. I felt like I was dying.” — P4*.

*“I started feeling dizzy in the morning. My husband told me I blacked out for hours.” — P 11*.

*“I started vomiting black liquid before anything else. It was like my insides were reacting instantly.” — P12*.

*“My saliva tasted like artificial sweetener*,* like saccharin. I’ve never tasted anything like it.” — P3*.

*“Our eyes became blurred; for two weeks we couldn’t see properly.” — P2*.

*“My eyes were burning so badly they gave me an injection just for the pain.” — P10*.

*“There was vision loss and shortness of breath—even while carrying people I could barely see through the smoke.” — P5*.

The short-term medical symptoms lasting days to weeks following the attack, participants continued to experience serious health problems. All survivors reported neurological symptoms, including tremors, confusion, or disorientation. Ophthalmologic issues persisted in 12 participants, while respiratory symptoms remained in 7. Gastrointestinal distress was reported by 6, along with sensory disturbances, temporary loss of consciousness, and involuntary urination. Notably, 2 participants also reported reproductive health complications. These ongoing symptoms disrupted daily functioning, prolonged recovery, and contributed to lasting trauma. Table 2.

*“If I try to smoke a cigarette now*,* just one puff makes me cough for an hour.” — P6*.

*“for the next two weeks I had tremors and couldn’t even control my hands” __ P13*.

*“It took my over 10 days until I could move myself*,* and until now I have nerve problems” __9*.

*“For 15–20 days after the attack I couldn’t see*,* but then it started improving slowly” __P4*.

*“. I passed out after few minutes*,* and I wake up after 7 hours in a different place*,* I found myself in a different place*,* in a hospital*,* and for the next 3 days I couldn’t concentrate*,* and then I realized that my kids are dead” P__9*.

### Long term medical symptoms

In the months and years following the attack, participants described a wide range of chronic and progressively debilitating conditions. The most commonly reported long-term symptoms were neurological and neuromuscular disorders, present in all participants. These included tremors, nerve pain, and coordination issues. Chronic respiratory problems were reported by 10 out of 14 survivors, while six participants reported symptoms suggestive of impaired immune function, including recurrent infections and prolonged recovery from common illnesses. Ophthalmologic complications persisted in 8, reproductive health disorders were mentioned by 3, and cardiologic issues by 4. Survivors also reported fatigue and weakness, gastrointestinal distress, and a variety of other symptoms, including caregiver-reported cognitive issues and developmental delays in children. Table 2. These long-term effects severely limited daily functioning, even years after the exposure. These narratives reveal a consistent pattern of multisystem chronic illness, in individuals who were previously healthy, and suggest a pattern of multisystem symptoms reported by participants. Most survivors explicitly attributed these conditions to the chemical attack, reinforcing the link between exposure and long-term health deterioration.

*“They get episodes—days where they shake, their nerves hurt. They’re always taking medication for it.” — P5.*

*“Even now, years later, I get tremors in my face, especially around my nose. It comes suddenly and really disturbs me.” — P6.*

*“I lost my eyesight, and my immune system is wrecked.” — P3.*

*“My son still has nerve inflammation. He walks and talks like an old man.” — P11.*

*“We noticed problems with urination. It’s like things stopped working right.” — P1.*

*“We lost so much weight after the attack—me and the kids. We looked like we were starving.” — P12.*

### Psychological impact

In addition to physical harm, the chemical attack left survivors with substantial psychological trauma. A significant majority reported symptoms consistent with anxiety, depression, or post-traumatic stress disorder (PTSD). Nightmares were described by 11 participants, and sleep disturbances and grief-related symptoms were reported by 8 each. These effects were closely linked to personal loss, mass casualties, displacement, and the erosion of community structures.

The trauma extended beyond the event itself, shaped by the chaos of emergency medical care, the psychological burden of mass burials, and the long-term fragmentation of families. Displacement functioned as both a physical and emotional rupture, often described as a point of no return. These layered experiences underline the urgent need for mental health support, including grief counselling and trauma-informed care, in post-conflict health interventions.

*“We went through a nightmare. I had a son and daughter—both lost in front of me. I still hear their voices at night.” — P10*.

*“When they took us to the hospital*,* we saw people lying on the floor*,* unconscious*,* children foaming at the mouth. My mind couldn’t process what I was seeing.” — P12*.

*“We were digging mass graves three meters deep. We were layering bodies. There was no time to say goodbye.” — P3*.

*“We were burying the dead quickly in collective graves. There were days when we buried 40 at once.” — P5*.

*“After the strike*,* we left into the unknown. We had no idea where we were going*,* just that we had to get away.” — P1*.

*“We were displaced to the north. We’ve only recently returned*,* and nothing feels the same.” — P2*.

*“We were displaced to the north. My sister left her children with me. She never came back.” — P13*.

**Table 2:** Prevalence of immediate, short-term, long-term, and psychological symptoms among survivors of the 2013 Sarin attack in Ghouta.

Theme	Subtheme	Frequency	Main results	Impact
Immediate symptoms	Excessive salivation & foaming	12	Response driven by instinct and community help; formal care was limited	Delayed response, confusion, and fear due to absence of structured alerts
	loss of consciousness	9		
	Muscle convulsions	11		
	ophthalmology symptoms	12		
	Respiratory Distress	11		
	Other	3		
Short term medical symptoms	GI distress	6	Symptoms were multisystemic and appeared rapidly after exposure	High toxicity confirmed; immediate collapse of physical function
	Neurologic symptoms	14		
	ophthalmology symptoms	12		
	Reproductive Health Disorder	2		
	Respiratory Distress	7		
	Sensory details	6		
	Temporary loss of consciousness	6		
	involuntary urination	5		
	Other	4		
Long term medical symptoms	cardiologic issues	4	Chronic symptoms persisted for years and disrupted daily life	Long-term disability, need for neurorehabilitation and monitoring
	chronic respiratory issues	10		
	GI distress	2		
	Symptoms of Immune dysfunction	6		
	Neurologic symptoms	14		
	ophthalmologic symptoms	8		
	Reproductive Health Disorder	3		
	weakness	5		
	other	7		
Psychological Impact	Self-reported psychological symptoms (e.g., anxiety, depressive symptoms)	12	The psychological trauma was deep and prolonged, rooted in loss and instability	Need for integrated mental health and grief support in recovery
	Sleep disturbances	8		
	Nightmares	11		
	Grief	8		

### Relationship between proximity, age, and severity of outcomes

Survivors’ testimonies shed light on how proximity to the chemical strike and age influenced the severity of outcomes. While the study did not apply formal statistical measures, strong narrative patterns suggest that closer distance to the impact site significantly increased both immediate and long-term health complications. In contrast, age appeared to have no protective effect—symptom severity was reported across all age groups.

Those within 10 to 30 m of the strike reported the most acute effects. Participant 1, only twenty meters away, described ***“witnessing seizures and foaming at the mouth”*** among others, and later experienced his own neuromuscular dysfunction and caregiver-reported cognitive decline. Participant 9, also in close range, recounted ***“near-total paralysis for ten days”*** along with lasting memory, vision, and cardiovascular complications. Participant 2, exposed within 30 m, noted respiratory impairment and long-term visual damage. Conversely, survivors farther from the epicenter experienced milder symptoms. Participant 4, who was about 100 m away, recalled shortness of breath and visual issues, but emphasized being outside the “deadly radius,” suggesting a localized exposure gradient. These narratives support the view that severity was dose-dependent, with closer proximity increasing toxic load and impairing escape capacity.

As for age, no clear correlation emerged. Participant 12, who has a 12-year-old daughter, describe how her daughter suffered vomiting, chronic fatigue, and psychological trauma. Meanwhile, 60-year-old Participant 10 reported visual and respiratory damage along with profound loss, both describing significant, ongoing health deterioration. Similarly, younger adults in their twenties and thirties also reported nerve damage, fertility issues, and chronic pain, undermining assumptions of age-based resilience.

**Fig. 2:**
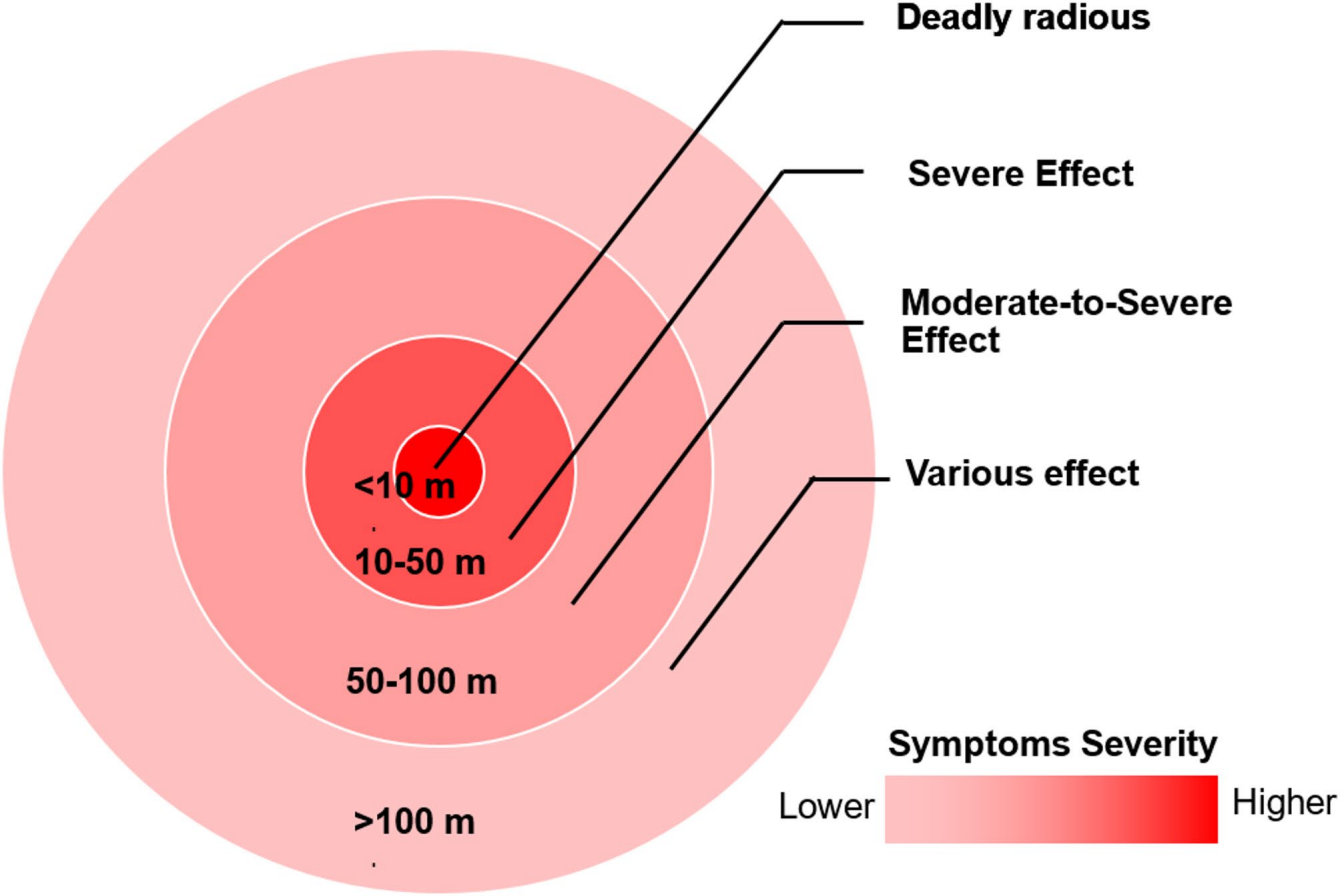
Severity of symptoms in relation to proximity to chemical attack location.

Figure 2 illustrates the relationship between distance from the chemical weapon epicentre and the severity of short- and long-term health effects among survivors. The innermost red zone (0–10 m) represents the deadly radius; no study participants were found within this proximity, nor did any report knowing someone who survived from this range, suggesting near-total lethality. The second circle (10–50 m) marks the severe exposure zone, where participants experienced the most intense acute symptoms (such as seizures, respiratory collapse, and unconsciousness) and severe long-term effects (including neuromuscular dysfunction and caregiver-reported cognitive decline). The third circle (50–100 m) corresponds to a moderate-to-severe exposure zone, where individuals reported short-term respiratory and visual symptoms alongside lasting, though less severe, chronic conditions. The outermost zone (> 100 m) reflects variable outcomes; while many experienced mild acute symptoms, some still reported persistent long-term issues, likely influenced by the direct action taken, individual vulnerability, or environmental shielding. Overall, the figure illustrates a clear proximity-dependent pattern of symptom severity, aligning with a dose-response relationship to sarin exposure.

## Discussion

The 2013 sarin gas attack on Ghouta represents one of the most severe chemical weapon incidents of the Syrian conflict, with significant and enduring public health implications. It was described by the United Nations as “the most significant confirmed use of chemical weapons against civilians since 1988,”^20^. Despite the magnitude of the attack, very little peer-reviewed research has been published, and to the best of our knowledge, no study has systematically documented the long-term health consequences of chemical weapon exposure during the Syrian war. In this retrospective study, we addressed this gap by investigating the lived experiences, short- and long-term medical symptoms, and psychological impact among civilian survivors of the Ghouta attack.

In the immediate aftermath of the attack, the response was largely shaped by local capacity and prior experience with chemical incidents^21^. Although formal emergency systems were limited, some health facilities in affected areas had pre-positioned atropine and basic supplies, reflecting a sense of preparedness among local healthcare workers following earlier chemical exposures^7^. Medical response during the acute phase relied heavily on community-based and volunteer efforts, with civilians and local responders transporting victims to overwhelmed field hospitals. At the same time, many residents adopted improvised protective measures, such as covering their faces with cloths soaked in vinegar or water. These actions were not based on evidence of chemical neutralization but rather reflected intuitive, locally held beliefs about protection and disinfection^22,23^.

Participants reported a range of persistent physical and psychological symptoms, including respiratory dysfunction, neurological complaints, and mental health disturbances, which they directly attributed to the sarin exposure. These included chronic cough, shortness of breath, chest pain, headaches, sensory deficits, memory problems, anxiety, and episodes of panic or depression—often coexisting and compounding one another. Many participants described a deterioration in their overall wellbeing, with symptoms interfering with daily tasks, family roles, and social interactions. The clinical symptoms reported by participants in our study are in strong agreement with those documented in the United Nations Mission report on the 21 August 2013 attack (A/67/997–S/2013/553), which concluded that “survivors reported a rapid onset of extreme breathing difficulties, eye irritation, foaming from the mouth, convulsions, and loss of consciousness.” The report also confirmed that biomedical samples from victims tested positive for sarin exposure, providing event-level confirmation of agent use and documenting a symptom profile consistent with nerve agent toxicity, which aligns with the patterns described in our qualitative findings^21^. The long-term neurological, respiratory, and psychological symptoms reported by participants are consistent with established toxicological evidence on organophosphate nerve agent exposure^24^. Sarin irreversibly inhibits acetylcholinesterase, leading not only to acute cholinergic crisis but also to persistent neuropsychiatric sequelae, autonomic dysfunction, and chronic respiratory complications documented years after exposure. Also, these findings are consistent with earlier case series from the Matsumoto and Tokyo subway sarin attacks, where victims reported long-term somatic and neuro-psychiatric symptoms; in the Tokyo cohort, 60–80% of survivors reported ocular and somatic symptoms at 10 years post-exposure and 35% met criteria for post-traumatic stress responses^25^. Additionally, our findings echo studies of Iranian veterans and Kurdish civilians exposed to sulfur mustard, where similar constellations of respiratory, neurological, and psychiatric symptoms were documented years or decades after exposure^26–29^. While toxicological mechanisms differ between sulfur mustard and sarin, the long-term outcomes appear to converge—highlighting the multi-systemic and enduring nature of chemical weapon injury. However, the Syrian case is further complicated by post-attack displacement, economic collapse, and the near-total breakdown of healthcare systems, which have left many survivors without diagnosis, treatment, or recognition^1,11,30^.

The results of our study showed that the psychological impact of the sarin attack was profound and enduring. Every participant described some form of emotional or psychological distress, often closely intertwined with their physical symptoms. Feelings of fear, helplessness, and anxiety were common, and many reported that these psychological burdens had exacerbated the severity of their chronic health problems. Nightmares, panic attacks, intrusive memories, and hypervigilance were frequently mentioned, alongside social withdrawal and emotional numbness. These findings are consistent with previous studies on chemical warfare survivors, which have documented elevated rates of PTSD, depression, and anxiety long after exposure^26,28^, and also similar reductions in quality-of-life scores and moderate depressive symptoms have been documented among Kurdish refugees resettled in Sweden thirty years after the initial attack^31^. In the Syrian context, where access to mental health care is virtually non-existent, the absence of recognition and support has further intensified the long-term psychological toll^32^.

Our findings also suggest that proximity to the epicentre of the attack may be a critical determinant of symptom severity. Participants who reported being closer to the impact zones consistently described more severe symptoms—ranging from acute respiratory failure and visual disturbances to long-term neurological and psychological sequelae. This pattern supports dose-dependent effects of sarin exposure, as noted in previous studies. In the Tokyo subway attack, for example, symptom severity and biomarker levels of sarin metabolites were significantly higher among individuals in closer proximity to the release site^25,33^. Similarly, after the Matsumoto attack, those residing nearest to the contaminated building experienced the highest mortality and greatest burden of chronic symptoms^25^. These findings align with toxicological models of nerve agent dispersal, which predict a steep exposure gradient based on distance, ventilation, and terrain^34^. Notably, the United Nations Mission report (A/67/997–S/2013/553) described similar spatial trends in Eastern Ghouta, where entire families residing near the impact sites were found deceased in confined spaces, often with no visible external injuries, suggesting rapid onset of fatal symptoms due to high-dose exposure. Such spatial clustering of fatalities near the epicentres reinforces the association between proximity and exposure severity in sarin-related mass casualty events^21^.

Access to appropriate healthcare emerged as a major challenge for all participants. Survivors reported years of living with unresolved symptoms—both physical and psychological—without receiving adequate diagnosis, treatment, or follow-up. These barriers mirror those faced by other chemical warfare survivors in conflict-affected settings, such as Halabja, where long-term care needs were similarly unmet^28^. However, in Syria, the systematic destruction of health infrastructure, ongoing displacement, and political denial of chemical attacks have compounded these difficulties— leaving many survivors without adequate diagnosis, follow-up, or access to appropriate care^35^.

This study offers one of the first systematic attempts to document the long-term health consequences of chemical weapon exposure among civilians during the Syrian war, drawing directly from the lived experiences of survivors. A key strength lies in its timing—conducted after the fall of the regime—which allowed for open, uncensored testimonies rarely accessible in previous years. The use of a retrospective descriptive design and survivor narratives provided a context-specific understanding of both medical and psychosocial outcomes. However, the study has limitations, including potential recall bias, survivor bias, and challenges in verifying clinical diagnoses due to the destruction/absence of medical records and lack of diagnostic follow-up.

Despite these limitations, our study underscores the urgent need for comprehensive, multidisciplinary care for sarin survivors. The persistence of symptoms ten years after exposure emphasises that acute treatment alone is insufficient. Long-term rehabilitation should integrate pulmonology, neurology, ophthalmology and mental-health services, as advocated for Halabja survivors. Community-based psychosocial support and recognition of survivor narratives are essential to address the chronic fear, social isolation and stigma highlighted by participants. International bodies and donor agencies must prioritise funding for chemical weapons survivors, support research into antidotes and neuroprotective therapies, and uphold mechanisms for accountability and reparations. Addressing the enduring health consequences of chemical weapon exposure is essential for informing long-term medical care, rehabilitation strategies, and public health preparedness which in turns contribute to the process of peacebuilding and transitional justice.

## Conclusion

This study provides qualitative insight into the long-term physical and psychological experiences of survivors of the 2013 sarin attack in Ghouta. Participants described persistent symptoms and lasting disruptions to daily life more than a decade after exposure.

While causal relationships cannot be established within this study design, the findings highlight a substantial self-reported burden of illness and unmet healthcare needs among survivors. Addressing these needs may be an important component of post-conflict health system recovery and long-term care planning. Incorporating survivor perspectives can contribute to more responsive medical, psychosocial, and policy interventions in similar contexts.

### Limitations

This study has several limitations inherent to retrospective qualitative research. Recall bias may have influenced participants’ accounts of symptoms and timelines, particularly given the long interval since exposure. Survivor bias is also likely, as the findings reflect the experiences of individuals who survived the attack and were available and willing to participate. In addition, long-term physical and psychological outcomes may be partially confounded by cumulative war-related trauma, displacement, and prolonged stress, which are difficult to disentangle from the direct effects of sarin exposure. The absence of systematic medical records and clinical verification further limits causal inference. These limitations were mitigated through purposive sampling of verified survivors, consistency across narratives, and cautious interpretation of findings as experiential rather than prevalence-based or causal. As no clinical examinations were performed, reported outcomes should be interpreted as symptom-based accounts rather than objective clinical signs.

Furthermore, the qualitative design and relatively small sample size limit the generalizability of the findings. The study does not aim to estimate prevalence or establish causal relationships, but rather to document lived experiences and reported health outcomes. Additionally, potential exposure misclassification cannot be excluded, as individual exposure levels were not objectively measured. These factors should be considered when interpreting the findings.

## Supplementary Information

Below is the link to the electronic supplementary material.

Supplementary Material 1

## Data Availability

The datasets generated and/or analysed during the current study are not publicly available due to confidentiality and security concerns but are available from the corresponding author on reasonable request.
