# Supplementary material for: Short and long-term health consequences of the 2013 Sarin attack in Ghouta, Syria: a retrospective descriptive study of civilian survivors
Source: Sci Rep. 2026 Apr 2;16:11379. doi: 10.1038/s41598-026-47135-z (PMC13049078; doi:10.1038/s41598-026-47135-z)
Supplement: Supplementary file 1 — Supplementary Material 1 [file 41598_2026_47135_MOESM1_ESM.docx]

| **Thematic Domain** | **Example Opening Prompts and Follow-up Probes** |
| --- | --- |
| **Exposure to Chemical Attack and lived experience** | • *Can you tell me, in your own words, what you remember about the day of the attack?*  • *Where were you at that time, and what did you notice happening around you?*  • *What made you realize that something unusual or dangerous was occurring?* |
| **Immediate medical response, treatment, and survival strategies** | • *What did you do once you realized you had been exposed?*  • *Did you or others around you try anything to protect yourselves or seek help?*  • *Can you describe how you reached medical care, if at all?* |
| **Immediate and Short-term medical symptoms** | • *What physical or psychological symptoms did you experience during or shortly after the attack?*  • *How soon did these symptoms begin, and how long did they last?*  • *Did these symptoms affect your ability to function in the days or weeks after the attack?* |
| **Long-term medical symptoms** | • *Have you experienced any ongoing health problems since the attack?*  • *How have these symptoms changed over time?*  • *In what ways have they affected your daily life or ability to work?* |
| **Psychological impact** | • *How did the attack affect you emotionally or psychologically?*  • *Do you still experience distress, fear, or memories related to the event?*  • *How have these experiences affected your relationships, sleep, or sense of safety?* |
